# Heterogeneous nuclear ribonucleoprotein K is a potential target for enhancing the chemosensitivity of nasopharyngeal carcinoma

**DOI:** 10.1515/biol-2022-0975

**Published:** 2024-10-30

**Authors:** Ming Yang, Zhaoyang Ke, Daji Wang

**Affiliations:** Department of Otorhinolaryngology, Shenzhen People’s Hospital (The Second Clinical Medical College, Jinan University, The First Affiliated Hospital, Southern University of Science and Technology), Shenzhen, Guangdong, 518020, China; Shenzhen Institute of Synthetic Biology, Shenzhen Institute of Advanced Technology, Chinese Academy of Sciences, Shenzhen, Guangdong, 518055, China

**Keywords:** nasopharyngeal carcinoma, chemotherapy resistance, chemosensitivity, heterogeneous nuclear ribonucleoprotein K

## Abstract

The resistance of tumor cells to chemotherapy drugs is a critical determinant in the recurrence and metastasis of nasopharyngeal carcinoma (NPC). Therefore, it is crucial to identify effective biotargets that can enhance the sensitivity of NPC cells to chemotherapy drugs. Heterogeneous nuclear ribonucleoprotein K (hnRNPK) plays a central role in regulating chemotherapy resistance across various tumor types. However, its specific function in NPC cells remains unclear. This study reveals that hnRNPK is overexpressed in NPC tissues while weakly expressed in normal nasopharyngeal tissues. The expression level of hnRNPK is negatively associated with NPC patient survival. Importantly, hnRNPK is a key inducer of chemotherapy resistance in NPC, as evidenced by the significant increase in NPC cell sensitivity to cisplatin following hnRNPK knockdown. Mechanistically, hnRNPK induces chemotherapy resistance in NPC cells by suppressing the activation of the Akt/caspase 3 pathway. In NPC tumor-bearing mice, hnRNPK knockdown enhances the efficacy of cisplatin chemotherapy. Consequently, this work identifies a potential target for enhancing the sensitivity of NPC cells to chemotherapy.

## Graphical abstract

**Figure:**
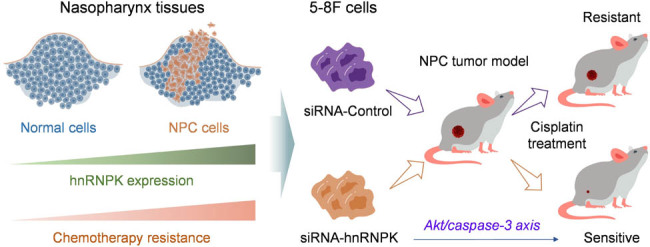

## 1 Introduction

Nasopharyngeal carcinoma (NPC) originates from the epithelial cells of the nasopharynx. It is a rare malignancy with distinct distribution patterns. Over 70% of new cases are reported in Southeast and East Asia, while the remaining cases are found in South-Central Asia and North and East Africa [1,2]. Early-stage or locally advanced NPC patients are primarily treated with chemotherapy [3,4]. Although chemo-radiotherapy has demonstrated promising results with a 5-year survival rate of 85–90% [5,6], approximately 8–10% of NPC patients experience tumor recurrence and metastasis. For recurrent NPC, multi-drug chemotherapy using platinum is the standard treatment. However, this method is limited by chemotherapy resistance. Therefore, it is crucial to understand the mechanisms of chemotherapy resistance in NPC to enhance therapeutic outcomes.

Diverse mechanisms contribute to the resistance of NPC to chemotherapy, including drug efflux, evasion of apoptotic processes, enhancement of DNA repair, activation of the epithelial-mesenchymal transition (EMT) pathway, and the presence of the Epstein-Barr virus (EBV) [6]. The continuous advancement of high-throughput sequencing technology has facilitated the identification of a growing repertoire of tumor-associated targets. For example, targeting specific proteins such as eukaryotic translation initiation factor 4E [7] and Bcl-2 [8] manifests as a strategic approach for inhibiting anti-apoptotic signaling pathways in NPC cells, thereby enhancing the effectiveness of chemotherapy. Furthermore, RAB37 [9] and circCRIM1 [10] induce the EMT process, leading to chemotherapy resistance in NPC. These potential targets provide valuable insights for clinical diagnosis, treatment, and drug development for NPC. The ongoing identification of additional biomarkers associated with chemotherapy resistance in NPC holds significant importance in understanding the recurrence and metastasis of NPC.

Heterogeneous nuclear ribonucleoprotein K (hnRNPK) serves a regulatory function within the heterogeneous nuclear ribonucleoprotein family. Recent studies highlight its crucial role in cancer progression [11,12,13]. hnRNPK plays a pivotal role in various cellular processes and is regulated by several kinases. hnRNPK suppression leads to a reduction in FLIP expression, simultaneously increasing the vulnerability of NPC cells to TRAIL-induced apoptosis [14]. Furthermore, hnRNPK activates the matrix metalloproteinase (MMP) 12 promoter, resulting in enhanced expression and enzyme activity of MMP12, which contributes to the migration and invasion of NPC cells [15]. Additionally, hnRNPK has been identified as a critical regulator of chemotherapy resistance in various malignancies. In ovarian cancer, hnRNPK interacts with mortalin to regulate ERp57 expression, thereby contributing to chemotherapy resistance to paclitaxel [16]. In acute leukemia, hnRNPK facilitates drug resistance by interacting with RNA 5-methylcytosine [17]. However, the relationship between hnRNPK expression and chemotherapy resistance in NPC remains unclear.

**Scheme 1:**
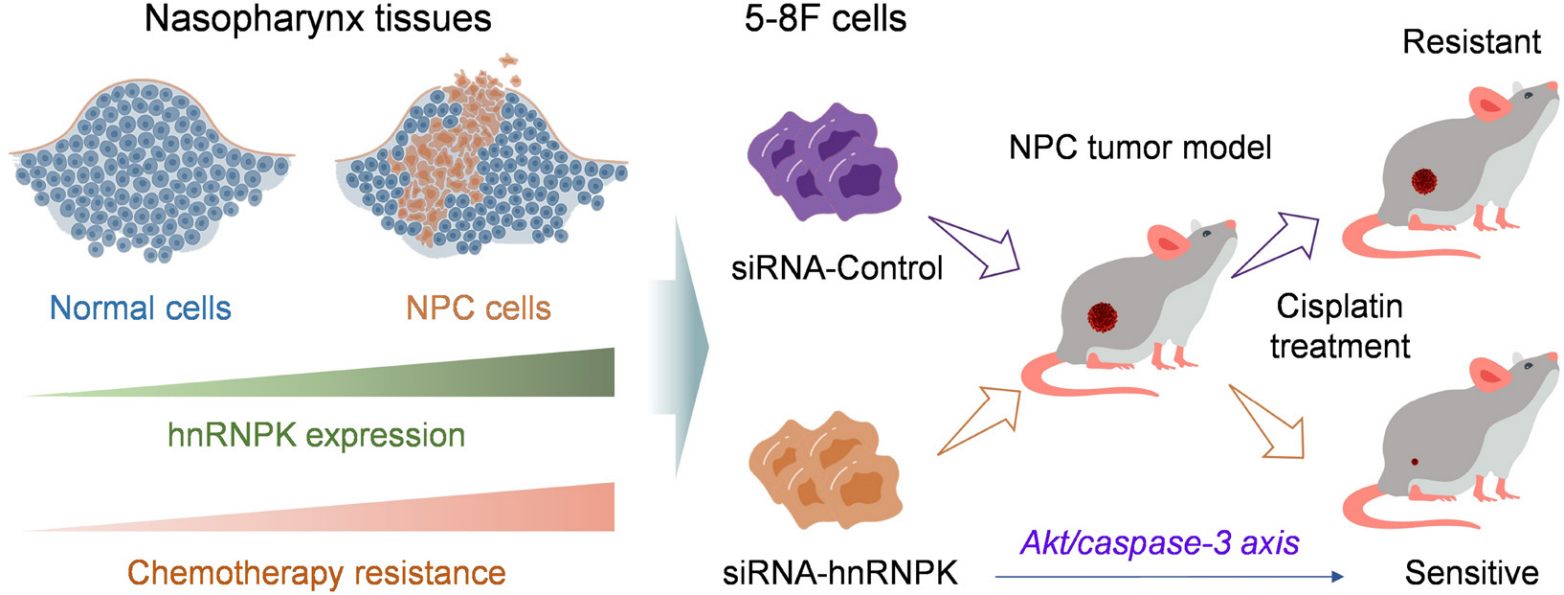
hnRNPK is a potential target for enhancing the chemosensitivity of NPC.

In this study, hnRNPK expression in clinical NPC samples was initially evaluated by using the GEPIA (http://gepia.cancer-pku.cn) and Oncomine (https://www.oncomine.org/). We analyzed the correlation between hnRNPK expression and the overall survival of NPC patients. Subsequently, we collected 10 NPC tissues and their corresponding nasopharyngeal tissues to verify the expression pattern of hnRNPK. hnRNPK expression was also investigated in various clinical human cell lines of NPC. Compared with other cell lines, 5–8 F cells showed higher hnRNPK expression, which was suitable for generating hnRNPK knockdown cells. As a result, inhibiting hnRNPK expression significantly enhanced the chemosensitivity of NPC cells to cisplatin and inhibited their migration. Moreover, Akt/caspase 3 signaling pathway was activated in 5–8 F cells after treatment with cisplatin, and this activation was more pronounced in 5–8 F cells with hnRNPK knockdown. Finally, we established tumor models by employing 5–8 F cells expressing different levels of hnRNPK to comprehensively evaluate the impact of hnRNPK expression on cisplatin chemotherapy (Scheme 1).

## 2 Materials and methods

### 2.1 Patients and clinical samples

Ten NPC tissues from newly diagnosed NPC patients and corresponding nasopharyngeal tissues were collected at Shenzhen People’s Hospital between August 2021 and December 2022 (Approval Number: SPH-2021-129-01). Consent was obtained from the patients and their families before specimen collection. The patient's age, sex, and pathological findings are recorded in Table 1. The NPC and adjacent tissues were removed using endoscopic forceps and placed into labeled 2 mL aseptic centrifuge tubes, which were then stored in liquid nitrogen tanks for preservation. The patient tissue samples were collected prior to treatment.

**Table 1:** Clinical information of NPC patients

Patients	Sex	Age	Stage	Chemo-therapy	Radio-therapy	HBsA*g* < 0.05 IU/mL	HIV-1/−2 < 1.0 S/CO	Anti-TP_C < 1.0 S/CO
#1	Male	49	T3N0M0 III	NO	NO	0	0.08	0
#2	Male	31	T3N2M0 III	NO	NO	0	0.12	0.04
#3	Male	44	T4N2M0 IVa	NO	NO	0	0	0.01
#4	Male	50	T3N0M0 III	NO	NO	0	0	0.03
#5	Female	54	T3N1M0 III	NO	NO	0	0	0
#6	Male	31	T3N3M0 IVa	NO	NO	0	0.04	0
#7	Male	51	T4N3M0 IVa	NO	NO	0.03	0	0
#8	Male	55	T4N2M0 IVa	NO	NO	0	0	0.06
#9	Male	70	T3N1M0 III	NO	NO	0.01	0.02	0
#10	Female	53	T3N1M0 III	NO	NO	0	0	0


**Informed consent:** Informed consent has been obtained from all individuals included in this study.
**Ethical approval:** The research related to human use has been complied with all the relevant national regulations, institutional policies and in accordance with the tenets of the Helsinki Declaration, and has been approved by the Ethics Committee of Shenzhen People’s Hospital (Approval Number: SPH-2021-129-01).

### 2.2 Cell culture and transfection

The clinical human NPC cell lines, C666-1, CNE1, CNE2, and 5–8 F, were cultured in HDMEM supplemented with 10% FBS. Normal nasopharyngeal epithelial cells (NP69) were cultured in an FBS-free KSFM medium with EGF1-53. hnRNPK knockdown in 5–8 F cells was achieved by transfection with siRNA-hnRNPK, delivered using the lentiviral vector pVSV-G (SyngenTech, China). The target sequences used for siRNA-hnRNPK were as follows: siRNA1-hnRNPK: 5′-CCUUAUGAUCCCAACUUUUTT-3′, siRNA2-hnRNPK: 5′-AAAAGUUGGGAUCAUAAGGTT-3′. The unmodified pVSV-G vector was utilized as the siRNA-Control. Transfected 5–8 F cells were selected and cultivated in the presence of ampicillin until a stable transfected cell line was established.

### 2.3 qPCR assay

TRIzol reagent (Invitrogen, USA) was used to extract total RNA of NPC cells. The total RNA was reverse-transcribed into cDNA using random primers. Subsequently, the qPCR assay was conducted using the ChamQ Universal SYBR qPCR Master reagent (Vazyme Biotech Co., Ltd, China) according to the manufacturer′s instructions. Table 2 provides information about the specific primers. β-Actin served as the housekeeping gene.

**Table 2:** qPCR primer sequences

Gene name	Primer sequence
hnRNPK	Forward: 5′-CAATGGTGAATTTGGTAAACGCC-3′
	Reverse: 5′-GTAGTCTGTACGGAGAGCCTTA-3′
β-actin	Forward: 5′-CATGTACGTTGCTATCCAGGC-3′
	Reverse: 5′-CTCCTTAATGTCACGCACGAT-3′

### 2.4 Western blots

The cells were lysed using a buffer containing inhibitors for proteases and phosphatases. Proteins were then separated using Sodium Dodecyl Sulfate PolyAcrylamide Gel Electrophoresis (SDS–PAGE) and transferred onto nitrocellulose membranes. Immunoblotting was performed on membranes. For the immunoblotting, several antibodies were utilized. These included anti-hnRNPK and anti-GAPDH from 4 A Biotech in China, anti-Akt, anti-phospho-Akt, anti-caspase 3, and anti-cleaved-caspase 3 from Cell Signaling Technology (USA).

### 2.5 Flow cytometry

For the evaluation of hnRNPK expression in 5–8 F cells with siRNA-hnRNPK or siRNA-Control were preincubated with an APC marked anti-hnRNPK antibody and tested using FACS Aria III (Becton, Dickinson and Company, USA). For cisplatin-induced apoptosis analysis, 5–8 F cells with siRNA-hnRNPK and siRNA-Control were treated with 5 μg/mL cisplatin for 24 h. Annexin V-FITC/PI staining was used to distinguish between apoptotic and live cells. Finally, the cells were tested using FACS Aria III.

### 2.6 Wound healing assay

About 5–8 F cells were cultured in 12-well plates until reaching 90% confluence. Streaks were meticulously generated in the monolayer culture using 10-µL pipette tips. Following the removal of suspended cells through washing procedures, the residual cells underwent treatment with HDMEM supplemented with 10% FBS. The migratory results were documented through photographic documentation conducted 24 h post-wounding.

### 2.7 Animal assays

Animal husbandry, handling, and experimentation were approved by the Institutional Animal Care and Use Committee of Shenzhen People’s Hospital (Approval Number: SPH-210804-0869). Female BALB/c nude mice, aged six to eight weeks, were obtained from SiPeiFu company (Beijing, China). Mice were housed under standard conditions with *ad libitum* access to sterile food and water and killed by CO_2_ asphyxiation followed by cervical dislocation unless otherwise stated. To establish the 5–8 F cell xenograft tumor model, mice were injected subcutaneously with 2 × 10^6^ siRNA-hnRNPK 5–8 F cells or siRNA-Control 5–8 F cells in 100 μL 1% PBS. When the size of the tumor reached approximately 200 mm^3^ (8 days after tumor implantation), the tumor model was intraperitoneally injected with 0.2 mg/kg cisplatin. The tumor volume was calculated according to the formula: *V* = 0.5 × *L* × *W* ^2^, where *L* represents the tumor length and *W* represents the tumor width. During the cisplatin treatment, body weight measurements were taken to evaluate drug toxicity.


**Ethical approval:** The research related to animal use has been complied with all the relevant national regulations and institutional policies for the care and use of animals, and has been approved by the Institutional Animal Care and Use Committee of Shenzhen People’s Hospital (Approval Number: SPH-210804-0869).

### 2.8 Pathological staining of tumor sections

Tumors or corresponding nasopharyngeal tissues were obtained from NPC patients or 5–8 F cells bearing mice. The tissues were fixed in a solution of 4% paraformaldehyde, followed by dehydration and embedding in paraffin. For histological analysis, the entire tumor tissues were serially sectioned and subsequently stained with hematoxylin and eosin. For proliferation and apoptosis analysis, Ki67 (Mreda, China) and TUNEL (KeyGEN BioTECH, China) staining was performed according to the manufacturer’s instructions.

### 2.9 Statistic methods

Statistical analysis of the data was conducted using GraphPad Prism 9 software (GraphPad Software, USA) and Origin 2024 (OriginLab, USA). To evaluate differences between the two groups, the unpaired two-tailed Student’s *t*-test was employed. For multiple comparisons, two-way ANOVA test was performed. Error bars represent the mean ± standard deviation (SD) calculated from a minimum of three independent experiments. Correlation analysis was evaluated using multiple linear regression and Spearman’s Rank correlation coefficient. Statistical significance was set at *p* < 0.05.

## 3 Results

### 3.1 hnRNPK expression analysis in NPC tissues

**Figure 1:**
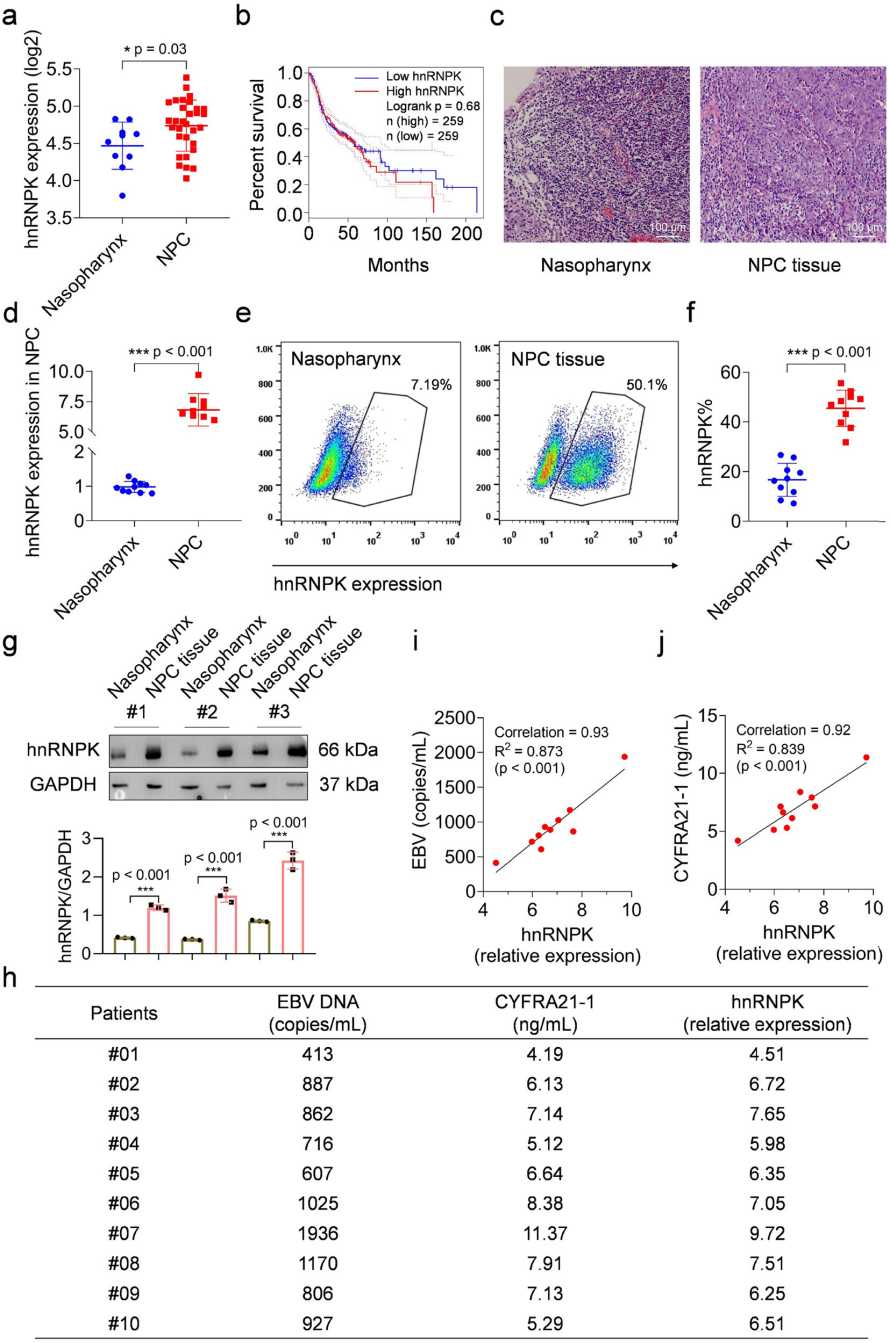
The expression of hnRNPK in NPC. (a) hnRNPK expression analysis in 10 normal nasopharyngeal tissues and 31 NPC tissues according to GEPIA and Oncomine databases. (b) The correlation analysis of hnRNPK expression and overall survival of NPC patients. (c) H&E staining for normal nasopharyngeal and NPC tissues. (d) qPCR assays for hnRNPK gene expression in NPC tissues. (e) and (f) hnRNPK expression of NPC samples was evaluated by flow cytometry. (g) Western blots for hnRNPK protein expression in NPC tissues. (h) EBV DNA (copies/mL) and CYFRA21-1 (ng/mL) in blood of NPC patients. (i) and (j) Correlation analysis of hnRNPK expression in NPC tissues and the levels of EBV DNA and CYFRA21-1 in blood of NPC patients.

The expression of hnRNPK in human NPC tissues was initially assessed using the GEPIA (http://gepia.cancer-pku.cn) and Oncomine (https://www.oncomine.org) databases. Figure 1a showed an analysis of 10 normal nasopharyngeal tissues and 31 NPC tissues. The expression of hnRNPK was significantly upregulated in NPC tissues compared to normal nasopharyngeal tissues. Meanwhile, we investigated the correlation between the hnRNPK expression and the overall survival rate of NPC patients. Figure 1b illustrated that higher hnRNPK expression in NPC was associated with a lower overall survival rate of patients.

Next, we measured hnRNPK expression in 10 NPC tissues and their corresponding normal nasopharyngeal counterparts. Normal nasopharyngeal tissues exhibited a typical mucosal structure, whereas the NPC tissues showed the presence of cancer nests. The NPC cells were characterized by their round or oval shape, vesicular nuclei, and abundant cytoplasm. Histologically, these tissues were diagnosed as undifferentiated non-keratinized NPC (Figure 1c). qPCR Results showed that the expression level of hnRNPK in NPC tissues was approximately 6.8 times higher than in normal nasopharyngeal tissues (Figure 1d). Additionally, we examined the protein expression of hnRNPK in NPC tissues. As shown in Figure 1e and f, the proportion of hnRNPK-positive cells in NPC tissues was significantly higher than in normal nasopharyngeal tissues. To further confirm this result, we utilized the normal nasopharyngeal tissues and NPC tissues from three patients to perform Western blots. The results further corroborated the elevated expression pattern of hnRNPK in NPC tissues (Figure 1g).

Two NPC-associated risk factors, EBV [18] and cytokeratin fragment antigen 21-1 (CYFRA21-1) [19,20], were observed to be significantly expressed in the blood of NPC patients (Figure 1h). To elucidate the association between hnRNPK expression and NPC risk, we conducted a correlation analysis between the mRNA level of hnRNPK in NPC tissues and the concentration of EBV DNA or CYFRA21-1 in the corresponding patient’s blood. Our findings revealed a significant correlation between hnRNPK expression and both EBV DNA and CYFRA21-1 levels, with correlation coefficients of 0.93 (EBV DNA) and 0.92 (CYFRA21-1), respectively (Figure 1i–j). Consequently, our analysis of both database information and actual tissue samples provides evidence for the upregulation of hnRNPK and its potential role in NPC development.

### 3.2 hnRNPK knockdown in human NPC cells

**Figure 2:**
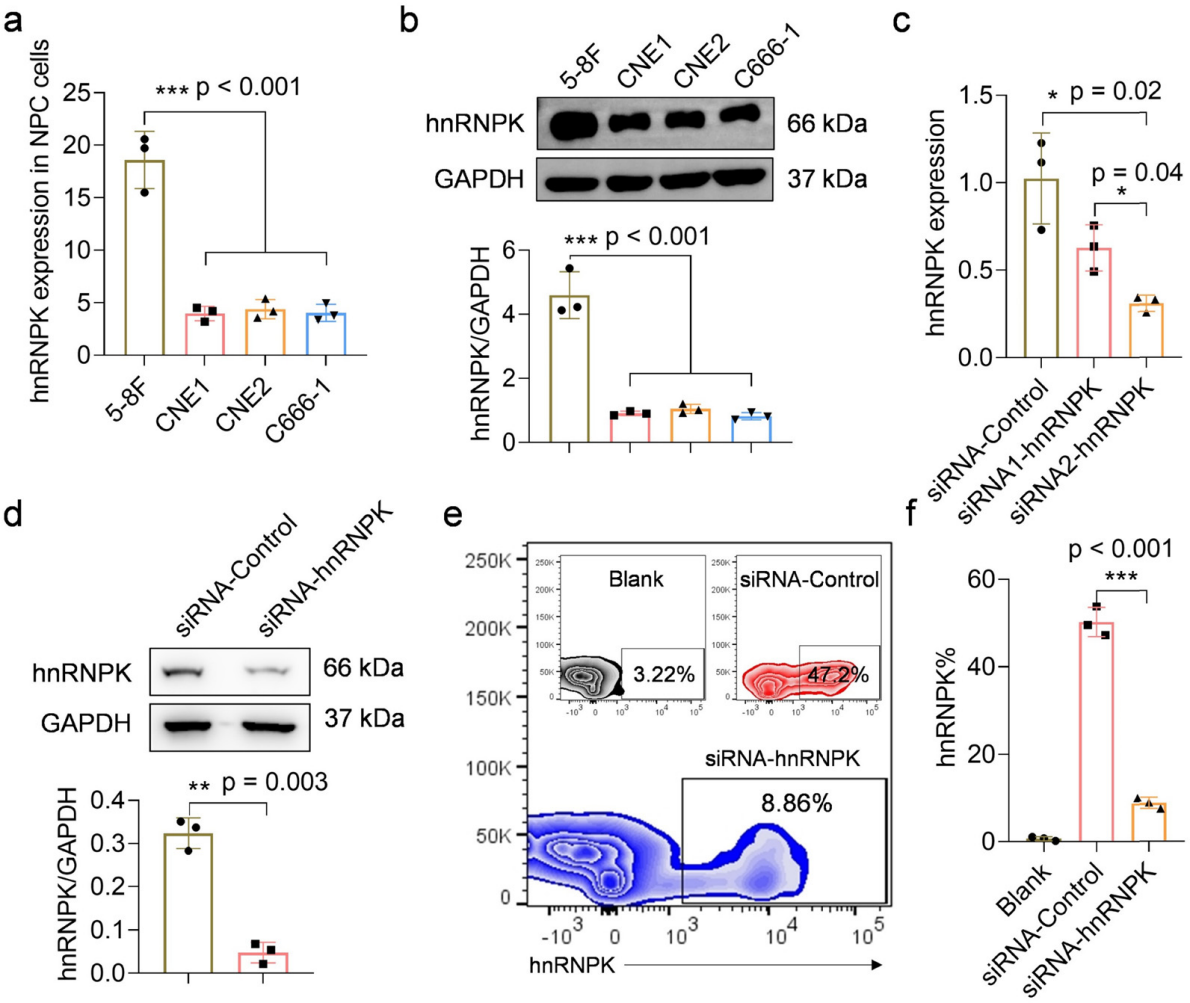
hnRNPK knockdown in clinical human NPC cells. (a) The expression of hnRNPK in NPC cell lines was detected by qPCR assay. (b) Evaluation of hnRNPK protein expression in NPC cell lines. (c) The knockdown efficiency of hnRNPK in 5–8 F cells was evaluated by qPCR assay. (d) The knockdown efficiency of hnRNPK in 5–8 F cells was evaluated by Western blots after ampicillin selection. (e) and (f) The knockdown efficiency of hnRNPK in 5–8 F cells was evaluated by flow cytometry after ampicillin selection. Blank represents siRNA-Control cells that are not labeled with anti-hnRNPK-APC antibody.

To investigate the relationship between hnRNPK expression and NPC development, we utilized siRNA transfection to achieve hnRNPK knockdown in human NPC cells. We first evaluated hnRNPK expression in several human NPC cell lines. Figure 2a showed that hnRNPK was differentially expressed in several human NPC cell lines, including 5–8 F, CNE1, CNE2, and C666-1. Specifically, hnRNPK expression in 5–8 F cells was approximately fourfold higher than that in the other three NPC cell lines. This finding was further confirmed by Western blots (Figure 2b). Thus, 5–8 F cells were selected to investigate the association between hnRNPK expression and pathology of NPC cells.

hnRNPK was knocked down in 5–8 F cells by using siRNA-hnRNPK. The sequences for the target of hnRNPK were meticulously designed as follows: siRNA1-hnRNPK, 5′-CCUUAUGAUCCCAACUUUUTT-3′ and siRNA2-hnRNPK, 5′-AAAAGUUGGGAUCAUAAGGTT-3′. We subsequently incorporated the sequences into the ampicillin-resistant lentiviral vector pVSV-G. The transfection efficiency of both siRNAs was evaluated using qPCR assays. Compared to siRNA-Control cells, hnRNPK expression was more significantly inhibited in the siRNA2-hnRNPK group compared to that in the siRNA1-hnRNPK group (Figure 2c). Therefore, siRNA2-hnRNPK was selected as the siRNA-hnRNPK for subsequent experiments. Finally, the transfected 5–8 F cells underwent selection in the presence of ampicillin until a stable transfected cell line was established. The knockdown efficiency of hnRNPK was evaluated using Western blots and flow cytometry. As shown in Figure 2d, we successfully generated stable hnRNPK knockdown 5–8 F cells. The expression of hnRNPK in siRNA-hnRNPK cells exhibited an approximate 75% reduction compared to siRNA-Control cells. Moreover, the proportion of hnRNPK-positive 5–8 F cells decreased by 40% following hnRNPK knockdown (Figure 2e and f).

### 3.3 hnRNPK knockdown enhances the chemosensitivity of NPC cells and inhibits their migration

**Figure 3:**
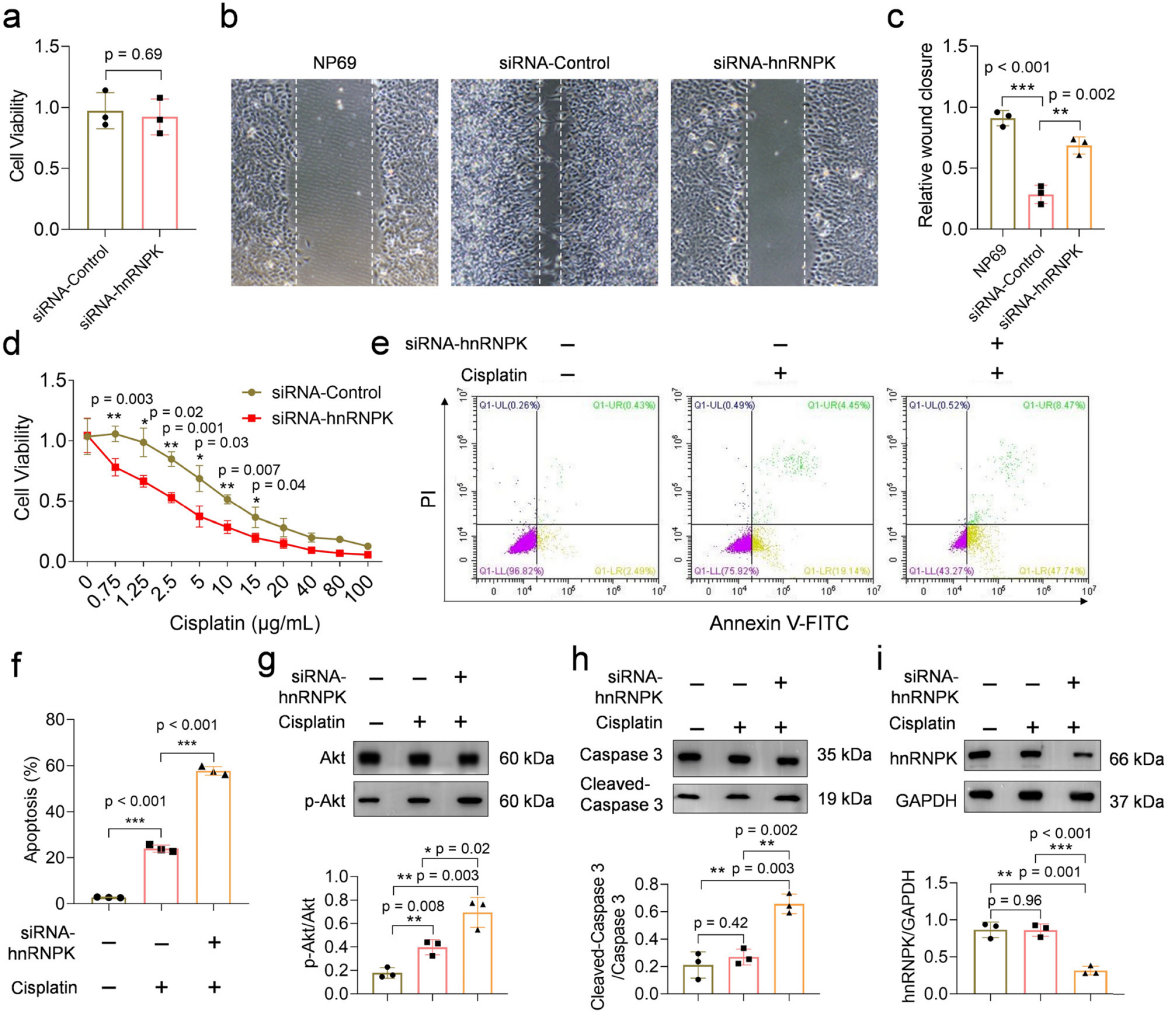
hnRNPK knockdown inhibits the migration and enhances the chemosensitivity of NPC cells. (a) CCK8 assay for detecting the effect of hnRNPK knockdown on cell viability. (b)–(c) The wound healing assays for NP69, siRNA-Control, and siRNA-hnRNPK cells. (d) CCK8 assay showed that knockdown of hnRNPK increased the sensitivity of 5–8 F cells to cisplatin treatment. (e) and (f) 5 μg/mL cisplatin induced higher apoptosis in siRNA-hnRNPK cells compared to siRNA-Control cells. (g)–(i) The activation of Akt/caspase 3 signaling pathway was detected by Western blots.

Many studies have demonstrated the association between aberrant hnRNPK expression and tumor progression [21,22,23]. Furthermore, hnRNPK is pivotal in determining the chemotherapeutic resistance of tumors. We next systematically investigated the relationship between hnRNPK expression level and the viability, migration, and chemotherapy resistance of 5–8 F cells. To investigate the impact of hnRNPK knockdown on the viability of NPC cells, we simultaneously cultured both siRNA-Control and siRNA-hnRNPK 5–8 F cells. Our results showed that hnRNPK knockdown had little effect on the viability of 5–8 F cells (Figure 3a). Notably, a positive correlation was observed between the migration capability and hnRNPK expression in 5–8 F cells, demonstrating that hnRNPK knockdown led to a significant inhibition of migration in 5–8 F cells (Figure b and c).

We subsequently conducted a systematic analysis of the correlation between hnRNPK expression and chemotherapy sensitivity in NPC cells. Cisplatin, recognized as an effective chemotherapeutic agent for NPC, was employed to evaluate the chemotherapy sensitivity of both siRNA-Control and siRNA-hnRNPK cells [24]. In Figure 3d, it was demonstrated that hnRNPK knockdown significantly enhanced the sensitivity of 5–8 F cells to cisplatin, particularly at lower concentrations from 0.75 to 15 μg/mL. This outcome implied that hnRNPK knockdown reduced the requisite amount of cisplatin to achieve the desired chemotherapy effect. To further elucidate this synergistic effect, flow cytometry was used to investigate cisplatin-induced apoptosis in 5–8 F cells. Figure 3e and f showed that 5 μg/mL cisplatin-induced approximately 23% apoptosis in siRNA-Control cells, whereas siRNA-hnRNPK cells exhibited an elevated apoptosis rate of approximately 56% at the same cisplatin concentration. Therefore, our findings demonstrate that high expression of hnRNPK enhances the resistance of NPC cells to chemotherapy drugs at the cellular level, while artificial attenuating hnRNPK expression improves the sensitivity of NPC cells to chemotherapy drugs.

Finally, we performed a preliminary analysis of alterations in apoptosis-related signaling pathways to elucidate the cell signaling mechanisms responsible for the increased sensitivity of siRNA-hnRNPK cells to cisplatin. The Akt/caspase 3 signaling axis is a classical pathway involved in governing cell apoptosis [25]. We observed that cisplatin significantly increased p-Akt expression in the siRNA-Control 5–8 F cells. In addition, cisplatin significantly upregulated the protein level of cleaved-caspase 3. Importantly, the protein expression of p-Akt and cleaved-caspase 3 induced by cisplatin was notably higher in siRNA-hnRNPK cells than in siRNA-Control cells (Figure 3g–i). These findings demonstrate that hnRNPK knockdown promotes cisplatin-induced apoptosis at the molecular level. The Akt/caspase-3 signaling axis emerges as a prospective pathway involved in hnRNPK-induced chemotherapy resistance in NPC cells.

### 3.4 hnRNPK knockdown enhances the antitumor effect of cisplatin in NPC tumor models

**Figure 4:**
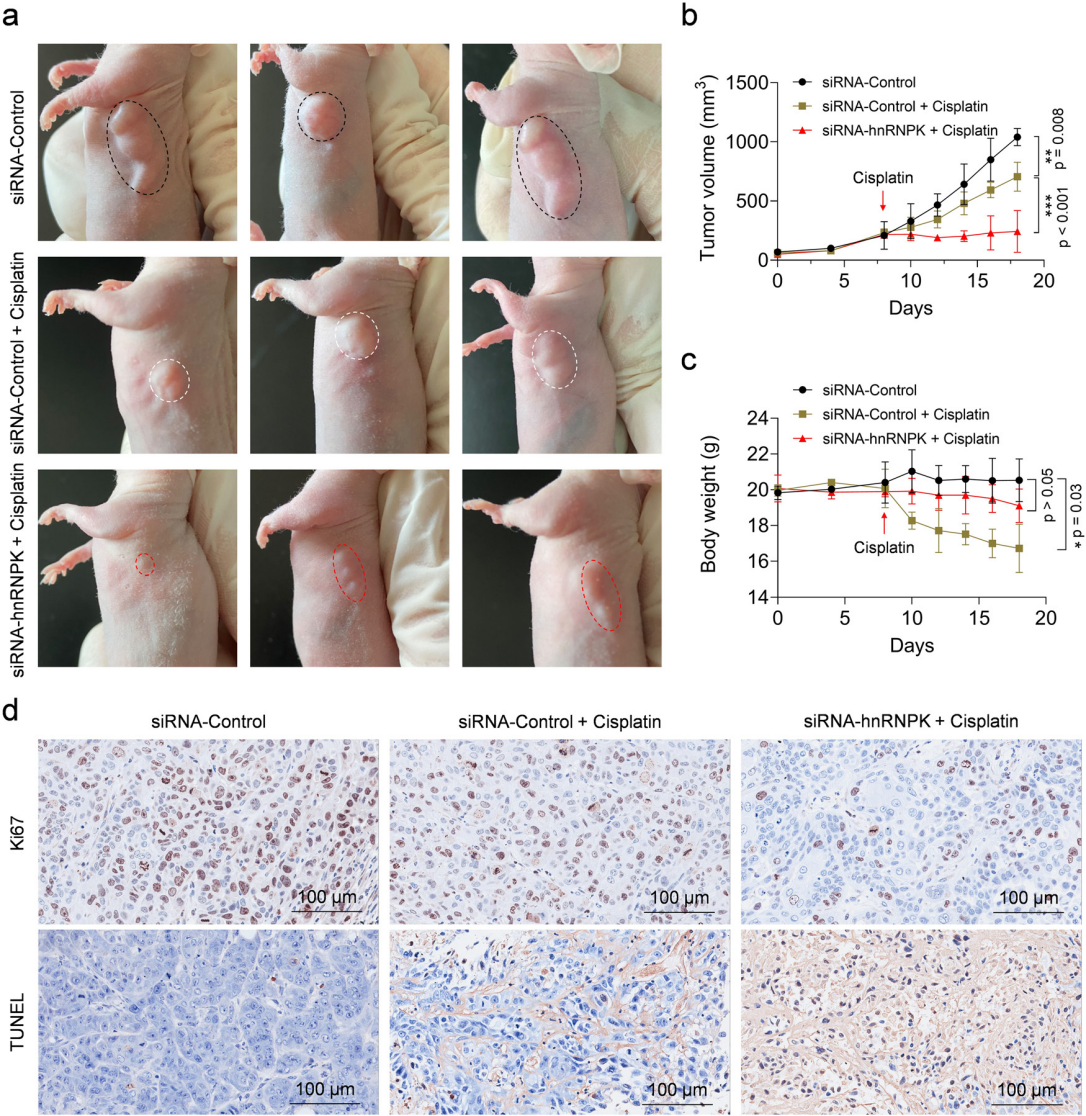
hnRNPK knockdown improves the antitumor effect of cisplatin in NPC tumor models. (a) The images of tumor morphology with cisplatin treatment in siRNA-hnRNPK and siRNA-Control groups. (b) The statistics of tumor volume after cisplatin treatment. (c) The effect of cisplatin on body weight of siRNA-hnRNPK cells- and siRNA-Control cells-bearing mice. (d) Ki67 and TUNEL staining for tumor sections.

To evaluate the effect of hnRNPK knockdown on the sensitivity of NPC cells to cisplatin treatment *in vivo*, BALB/c nude mice bearing siRNA-Control or siRNA-hnRNPK 5–8 F cells were used as tumor models. Once the tumor size reached approximately 200 mm^3^, the tumor models were administered with an intraperitoneal injection of 0.2 mg/kg cisplatin. As depicted in Figure 4a, tumor growth was completely inhibited within 10 days following cisplatin treatment in the siRNA-hnRNPK group. In comparison, 0.2 mg/kg cisplatin also significantly impeded the growth of siRNA-Control tumors, but its antitumor effect was unsatisfactory and the tumors remained large. Based on the tumor growth curve, the growth of siRNA-hnRNPK tumors was completely arrested after initiating 0.2 mg/kg cisplatin treatment at day 8, while cisplatin had little effect on siRNA-Control tumors, which continued to grow rapidly (Figure 4b). These results further demonstrated that knockdown of hnRNPK significantly improved the sensitivity of NPC cells to cisplatin treatment. we next observed changes in body weights of the tumor models following cisplatin administration. As shown in Figure 4c, the administration of cisplatin at a dosage of 0.2 mg/kg demonstrated notable systemic toxicity in mice; however, the weight loss observed in the siRNA-hnRNPK group was less pronounced compared to the siRNA-Control group. Thus, hnRNPK knockdown may enhance the tumor killing effect of cisplatin and promote the recovery of physiological function in mice.

Finally, the tumors were stained with Ki67 and TUNEL to evaluate the effects of cisplatin on proliferation and apoptosis of 5–8 F cells with different expression levels of hnRNPK. Figure 4d shows that cisplatin inhibited the proliferation of siRNA-Control cells, with a further reduction observed in the siRNA-hnRNPK group. In contrast, cisplatin triggered a substantial degree of apoptosis in siRNA-Control cells. The apoptosis induced by cisplatin was notably more pronounced in the siRNA-hnRNPK group. These results suggest that suppressing hnRNPK expression can reduce the required dosage of cisplatin, thereby attenuating its adverse effects while preserving comparable chemotherapeutic efficacy.

## 4 Discussion

Disease relapse in NPC is predominantly characterized by distant metastasis, affecting approximately 70% of NPC patients and ultimately leading to cancer-specific mortality [26,27]. Platinum-based agents are typically employed as the primary treatment for this subset of NPC patients. The resistance exhibited by tumor cells to these chemotherapy drugs significantly influences the recurrence or metastasis of NPC. Many strategies to overcome drug resistance in NPC are proposed. For instance, combination therapies are highly favored as a strategy to prevent the emergence of drug-resistant tumors. Inhibition of high mobility group box 1 (HMGB1) by glycyrrhizin significantly impedes the DNA binding ability of HMGB1, thereby enhancing the efficacy of chemotherapy [28]. The expression of multidrug resistance-associated (MDR) ABC transporters, specifically ABCB1, ABCG2 and ABCC1A, is a primary contributor to chemoresistance. The efficacy of overcoming MDR activity in preclinical models has been demonstrated through the combination of ABC transporter inhibitors with chemotherapeutic drugs [29]. Despite the development of third-generation ABCB1 inhibitors, clinical attempts to directly inhibit ABCB1 activity have proven unsuccessful. Furthermore, combination therapies inevitably increase the side effects. Therefore, it is crucial to identify specific biotargets that can enhance the sensitivity of NPC cells to chemotherapeutic drugs. In this study, we reported hnRNPK as a key target that increased the sensitivity of NPC cells to cisplatin. Compared to normal nasopharyngeal epithelial tissues, NPC tissues exhibited a substantial increase in hnRNPK expression. This augmented hnRNPK expression was positively correlated with the migration ability of NPC cells. However, the inhibition of hnRNPK expression did not cause direct apoptosis in NPC cells. Our findings demonstrated that knocking down hnRNPK increased the sensitivity of NPC cells to low-dose cisplatin treatment. Conversely, when exposed to high concentrations of cisplatin, extensive cell death ensued. Notably, hnRNPK knockdown exhibited no significant impact on the chemosensitivity of NPC cells under these conditions, demonstrating that inhibiting hnRNPK expression reduced the required dosages of cisplatin while maintaining equivalent chemotherapy effects. Toxicity following chemotherapy remains a relevant concern [30,31]. Thus, the strategic targeting of hnRNPK emerges as a promising and innovative approach for improving the sensitivity of NPC cells to chemotherapy.

The primary role of hnRNPK is its involvement in cell proliferation and migration, both of which are essential processes for tumor growth and progression [32,33,34]. The examination of hnRNPK expression patterns across diverse tumor types has provided valuable insights. For instance, hnRNPK expression is frequently upregulated in neoplasms such as gastric cancer, hepatocellular carcinoma, and breast carcinoma [35,36], implying its contribution to the aggressive nature and rapid proliferation of these tumors. Critically, as a biomarker of tumor apoptosis induced by chemotherapy, hnRNPK promotes metastases in tumors by up-regulating MMP [37], which may explain the overexpression of hnRNPK in drug-resistant tumors. On the other hand, hnRNPK has been found to be downregulated in certain tumors. This downregulation suggests that hnRNPK may play a potential suppressive role in the development of these specific malignancies [38,39]. The exact mechanisms underlying the aberrant expression pattern of hnRNPK in different tumors are still largely unknown and require further investigation. Interestingly, according to the statistical results of the database, hnRNPK is overexpressed in clinical NPC tissues compared with normal tissues, suggesting its potential oncogenic function. However, hnRNPK knockdown exhibits no discernible impact on cell viability but exerts a notable inhibitory effect on the migration of 5–8 F cells in this study. This migration promotion ability of hnRNPK is also well demonstrated in recent studies [40,41]. hnRNPK promotes the metastasis of tumor cells by interacting with some transcription factors or long noncoding RNAs such as prospero-related homeobox 1 [42] and LINC00941 [38]. Besides, the role of hnRNPK in chemotherapy resistance of NPC has received limited attention and remains unexplored. For the first time, we demonstrate that inhibiting hnRNPK expression considerably enhances the chemosensitivity of NPC, presenting a novel and effective strategy for combating chemotherapy resistance in NPC.

Akt signaling plays a critical role in regulating various biological processes such as cell metabolism, cell cycle, and angiogenesis [43,44]. Additionally, it is closely associated with tumorigenesis, metastasis, and drug resistance [45,46]. Therefore, Akt is considered a potential target for cancer treatment. Specifically, the axis of the Akt/caspase 3 pathway is necessarily involved in cell apoptosis [47]. In this study, we observed that cisplatin treatment triggered the activation of Akt/caspase 3. Interestingly, our findings reveal that hnRNPK knockdown amplified this activation, thereby heightening the sensitivity of NPC cells to cisplatin treatment. Consequently, hnRNPK knockdown potentially enhanced the efficacy of cisplatin chemotherapy by promoting Akt/caspase 3 apoptotic signaling. These results demonstrate that hnRNPK is a negative regulator upstream of the Akt/caspase 3 signaling axis, and blocking hnRNPK further enhances cell apoptosis via Akt/caspase 3 activation. Nevertheless, the specific mechanism that hnRNPK modulates the activation of the Akt/caspase 3 pathway through direct or indirect approach, remains to be conclusively determined, pending additional experimental evidence. Besides, several signaling pathways such as Wnt/β-catenin [42], p53 [40], and Hippo [48] are extensively involved in the oncogenic functions of hnRNPK. Further research is essential to elucidate the regulatory network both upstream and downstream of hnRNPK, which is crucial for the development of drugs that target hnRNPK.

In conclusion, we identified a novel association between hnRNPK expression and chemotherapy resistance in NPC cells both *in vitro* and *in vivo*. Notably, suppressing hnRNPK expression resulted in a substantial improvement in the sensitivity of NPC cells to cisplatin at low doses. Thus, targeting hnRNPK may offer a promising approach to overcoming chemotherapy resistance in NPC.
